# A ”Clickable” Probe for Active MGMT in Glioblastoma Demonstrates Two Discrete Populations of MGMT

**DOI:** 10.3390/cancers12020453

**Published:** 2020-02-14

**Authors:** Sudhir Raghavan, David S. Baskin, Martyn A. Sharpe

**Affiliations:** Department of Neurosurgery, Kenneth R. Peak Brain and Pituitary Tumor Center, Houston Methodist Hospital, 6670 Bertner Ave, Houston, TX 77030, USA; dbaskin@houstonmethodist.org

**Keywords:** MGMT, glioblastoma, click chemistry, cancers, temozolomide, DNA repair, drug resistance

## Abstract

Various pathways can repair DNA alkylation by chemotherapeutic agents such as temozolomide (TMZ). The enzyme O^6^-methylguanine methyltransferase (MGMT) removes O^6^-methylated DNA adducts, leading to the failure of chemotherapy in resistant glioblastomas. Because of the anti-chemotherapeutic activities of MGMT previously described, estimating the levels of active MGMT in cancer cells can be a significant predictor of response to alkylating agents. Current methods to detect MGMT in cells are indirect, complicated, time-intensive, or utilize molecules that require complex and multistep chemistry synthesis. Our design simulates DNA repair by the transfer of a clickable propargyl group from O^6^-propargyl guanine to active MGMT and subsequent attachment of fluorescein-linked PEG linker via ”click chemistry.” Visualization of active MGMT levels reveals discrete active and inactive MGMT populations with biphasic kinetics for MGMT inactivation in response to TMZ-induced DNA damage.

## 1. Introduction

DNA alkylating agents such as temozolomide (TMZ) are essential first-line chemotherapeutic agents used to treat several cancers such as glioblastomas (GBM) and melanomas [1]. These drugs mediate their cytotoxicity by forming covalent DNA adducts. TMZ is a blood-brain barrier permeable prodrug that gives rise to the highly reactive methylating agent diazomethane, which reacts with multiple nucleophilic sites on DNA [2]. Among its targets, diazomethane methylates the N^3^-position of adenine and the N^7^ and O^6^ positions of guanine, produces up to 13 different DNA base adducts. Of these adducts, O^6^-methylguanine (O^6^MeG), accounts for <8% of alkylation products, but is the primary route for induction of apoptosis, inducing futile cycling of the DNA mismatch repair pathway and causing DNA double-strand breaks [2,3]. 

TMZ is used as part of the Stupp protocol for the treatment of GBM [4]. The Stupp treatment protocol has led to significant survival improvements (2-year-survival increasing from 10.4% to 26.5%), but comes with high toxicity and the eventual emergence of resistance, often limiting usage. DNA alkylating agents such as CCNU are also part of treatment regimens for patients resistant to TMZ [5,6]. Resistance to TMZ and other alkylating agents is usually the result of upregulation of one or more DNA repair pathways, down-regulation of a pro-apoptotic signaling pathway, or due to drug-resistant phenotypes which have an innate upregulation of DNA repair pathways due to reactive oxidative stresses [7,8]. 

O^6^-methylguanine-DNA-methyltransferase (MGMT, also denoted AGT) is a DNA repair protein that directly removes alkyl adducts from O^6^-guanine and O^4^-positions of thymine. MGMT uses a conserved cysteine thiol (Cys145) in its active site to remove alkyl groups, forming an alkyl thioether bond in the process [9]. This process permanently inactivates MGMT, thus MGMT is termed a suicide enzyme. After inactivation there is an increase in the rate at which MGMT undergoes ubiquitination and then proteasomal degradation [10]. Tumors often overexpress MGMT, especially after exposure to alkylating agents during chemotherapy. The therapeutic effect of alkylating agents is inversely correlated to MGMT levels and depletion of MGMT protein levels is being explored as an attractive therapeutic strategy [11]. 

Most available methods that measure MGMT levels and activity are indirect and utilize complicated multi-step processes. One approach measures the methylation of the CpG gene promoter for the MGMT gene using methylation-specific PCR [12]. Methylation of the promoter sequence is indicative of low MGMT levels. However, this is an indirect measurement of MGMT levels, with only a modest correlation between MGMT activity and DNA methylation status that limits its predictive ability to identify resistant cancers [13]. An alternative approach proposed by Nagel et al. uses an ELISA-based assay to quantify MGMT [14]. This is a multi-step process that measures the total amount of MGMT in cells but does not distinguish between active and inactive MGMT. 

A third approach to measure MGMT levels involves using radioactive trackers such as O^6^-[^3^H]methyl guanine wherein tracking the transfer of the radioactive methyl group to the MGMT protein could be correlated to MGMT activity, though the resulting correlation was not linear with time [15]. Another radioactive probe that has been used to determine MGMT levels include ^32^P-labeled or fluorophore-labeled dsDNA probes containing O^6^MeG. This approach utilizes MGMT to repair the dsDNA, which upon repair generates a restriction enzyme cleavage site which can be detected by gel electrophoresis of DNA fragments [16,17]. Recently, a more straightforward fluorescent-based method for detection of MGMT was described by Kool et al. wherein they designed a chemosensor that contained both a fluorophore and a quencher which were separated by MGMT activity, leading to activation of the fluorophore and a light-up response of up to 55-fold [18].

While the above methods can detect/measure MGMT activity, they either include radioactive intermediates, or are multi-step processes requiring cell lysis and separation of components by gel electrophoresis, or involve elaborate synthetic routes to generate the components involved. Among the few reported fluorescence-based assays for MGMT measurement, Tintoré et al. described the use of aptamers containing an O^6^-MeG lesion, a fluorophore, and a quencher. Repair by MGMT decreases the distance between the fluorophore, thereby reducing the fluorescence, with observable, but small (<2-fold) signal changes [19]. Another fluorescence-based assay using an O^6^-benzylguanine (O^6^BG) mimic was reported by Robinson et al. where incubation with MGMT transferred the fluorophore to the protein [20]. The labeled protein fractions from whole-cell lysates were quantified after separation of the unreacted probe by thin-layer chromatography.

We recently reported a clickable fluorescence-based MGMT probe that transfers a propargyl moiety to MGMT from O^6^-propargyl guanine (O^6^PGG) [7]. The alkyne group transferred to MGMT undergoes a click reaction in the presence of copper and ascorbic acid with a subsequently introduced high molecular weight (~5000 Da) PEG which contains an azide group (click chemistry handle) at one end and FITC at the other (azido-PEG-FITC). Here we present methodological details of detecting and analyzing MGMT using our clickable linker and O^6^PGG in GBM cells. Analysis of this data obtained by incubating GBM cell lines provides valuable insights into MGMT trafficking and dynamics in GBM cells. 

### 1.1. Design of Fluorogenic Clickable MGMT Probes

The most common in vivo substrate of MGMT is O^6^-methylated DNA, with the resultant transfer of the methyl group from the O^6^-position of DNA to the Cys145 thiol of MGMT. Our probe contains a propargyl group that mimics the small size of the methyl group in *O*-methylated DNA, while simultaneously providing an alkyne handle that can be functionalized for visualization. Upon binding to MGMT the propargyl group from O^6^-propargylguanine is transferred to the Cys145 thiol of MGMT and generates an inactive propyne-MGMT adduct. The propyne-MGMT adduct can be easily visualized, using click chemistry, after coupling to a fluorescently-labeled azido-PEG-FITC. The synthesis of O^6^-propargylguanine had previously been described, before the usage of ‘click chemistry’ for labeling [21]. Cys145-adducts are found at the bottom of the long substrate channel in MGMT, and this imposes steric constraints on potential labeling groups. We chose to use a long, flexible, bifunctional linking agent, which was commercially available, to allow visualization of the propyne-MGMT adduct. Polyethylene glycol (PEG) based polymers are large amphiphilic molecules that are highly hydrated and flexible. Amino-PEG_5000_-azide is a bifunctional linker with an amine group that is easily coupled to the fluorophore FITC, forming a PEG-linked dye, azido-PEG-FITC. The large molecular weight of PEG assists in rapid and easy purification of azido-PEG_5000_-FITC from the reaction mixture by the use of ultracentrifugal filters or dialysis. 

Upon treating the propyne-MGMT adduct with azido-PEG-FITC/Cu(I), the azide/propyne groups undergo click coupling thereby labeling MGMT with a fluorophore. 

### 1.2. Synthesis of Fluorogenic Probes

**Scheme 1:**
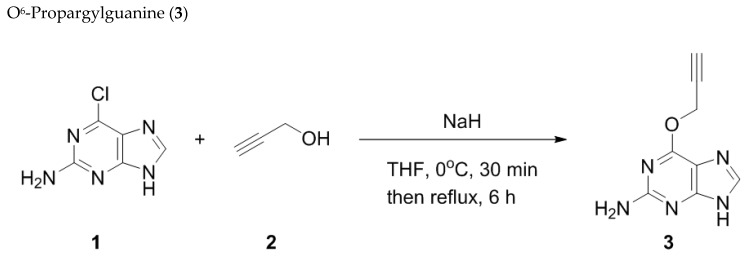
Synthesis of O^6^-propargylguanine (O^6^PGG).

**Scheme 2:**
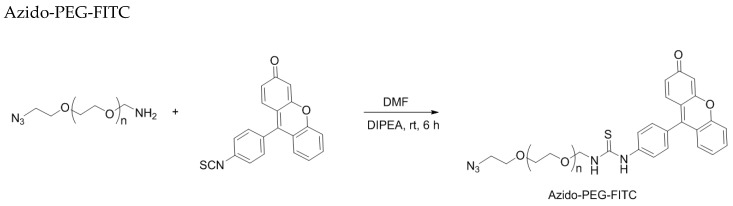
Synthesis of Azido-PEG-FITC.

O^6^PGG was synthesized using literature reported methods [21] by displacement of 6-chloropurine with deprotonated propargyl alcohol followed by chromatographic purification, (Scheme 1, Materials and Methods). Commercially available amino-PEG_5000_-azide was reacted with fluorescein isothiocyanate to provide the azido-PEG-FITC conjugate. The PEG-conjugate could be easily purified either by dialysis or ultracentrifugation followed by lyophilization (Scheme 2, Materials and Methods). 

The procedures used in the synthesis of the MGMT-probe have been designed to be performed in any biochemical/molecular biology lab and do not require any specialized organic chemistry experience. 

## 2. Results

### 2.1. Measuring Levels of Active MGMT in GBM Cells

**Figure 1:**
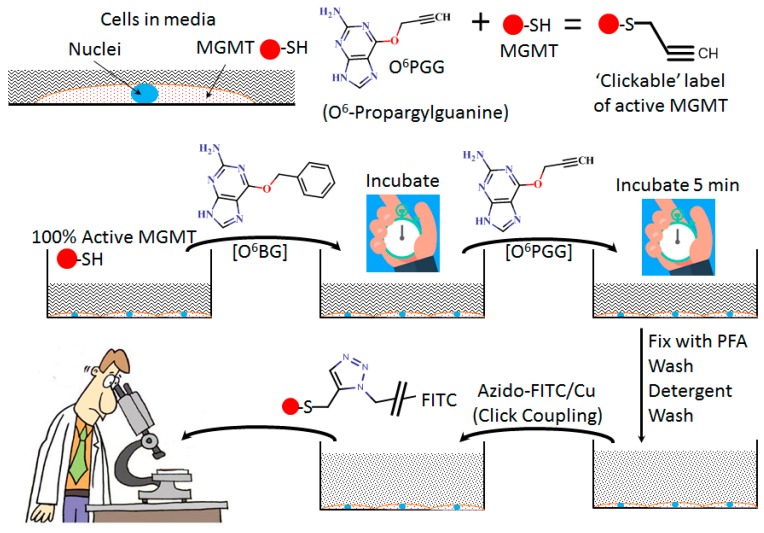
Cartoon depicting methodology used for the detection of active O^6^-methylguanine methyltransferase (MGMT) using O^6^PGG and Azido-PEG-FITC in glioblastomas (GBM).

**Figure 2:**
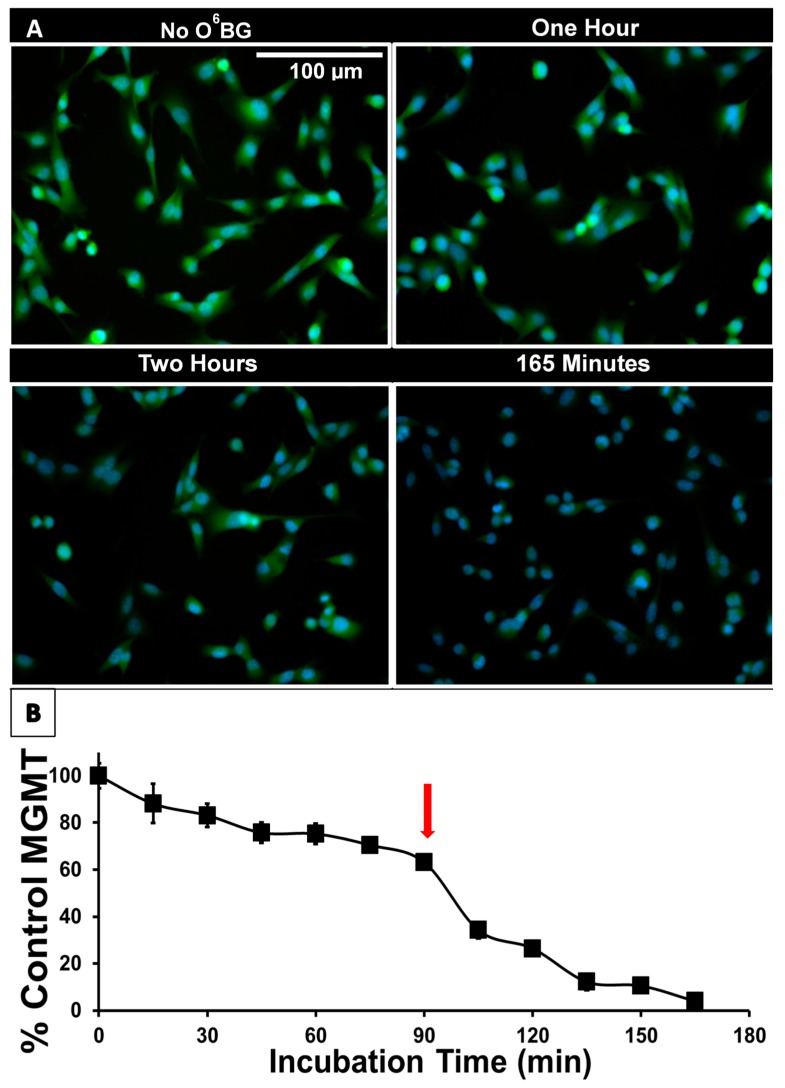
(**A**) Reduction of active MGMT upon incubation of GBM157 cells with 10 µM O^6^BG visualized with O^6^-propargyl (O^6^PGG) clicked with azido-PEG-FITC. (**B**) Quantification of fluorescence intensity shows a sharp reduction in active MGMT levels after about 90 min, indicated by the red arrow.

The general procedure used to determine the levels of active MGMT in GBM157 GBM cells is shown in the cartoon in Figure 1. These cells were incubated with O^6^BG, an MGMT inhibitor, for varying durations (0–165 min, see Figure 2), and then O^6^PGG was added to the standard culture medium. After further incubation, cells were fixed with paraformaldehyde. Cells were then labeled using the azido-PEG-FITC dye (adding 100 µM to aspirated slightly ”wet cells”) and copper-catalyzed click chemistry. Cells were then visualized using fluorescence microscopy. The nuclei were stained with DAPI.

### 2.2. Labelling of MGMT in GBM Cells and Reduction in Labeling by Incubation with TMZ

Figure 2A shows the labeling of GBM157 cells with O^6^PGG and azido-PEG-FITC. Control cells show high levels of labeled, i.e., active, MGMT present in the cytosol and nucleus. Nuclear colocalization of MGMT is shown in detail in Supplementary Figure S1. A time-dependent decline of active MGMT levels could be observed upon incubating the GBM157 cells with the 10µM O^6^BG followed by labeling. Quantifying the data (Figure 2B) shows a gradual reduction of active MGMT levels compared to control until about 90 min of incubation, at which time a sharp reduction in active MGMT levels is observed. The biphasic kinetics for the inactivation of MGMT by O^6^BG was unexpected and, to our knowledge, has not been reported before. 

The kinetics for active MGMT levels also seemed to indicate that there are two distinct pools of active MGMT in GBM157. We there undertook a further series of experiments to examine the properties of MGMT inactivation by the DNA alkylator TMZ.

A similar reduction in MGMT labeling by O^6^PGG/azido-PEG-FITC upon pre-treating cells with O^6^BG was observed with four additional primary GBM cell lines (GBM111, GBM115, GBM133, and GBM175) and is shown in Supplementary Figure S2.

### 2.3. MGMT Levels Increase Following TMZ Treatment, but Active MGMT Levels Fall

**Figure 3:**
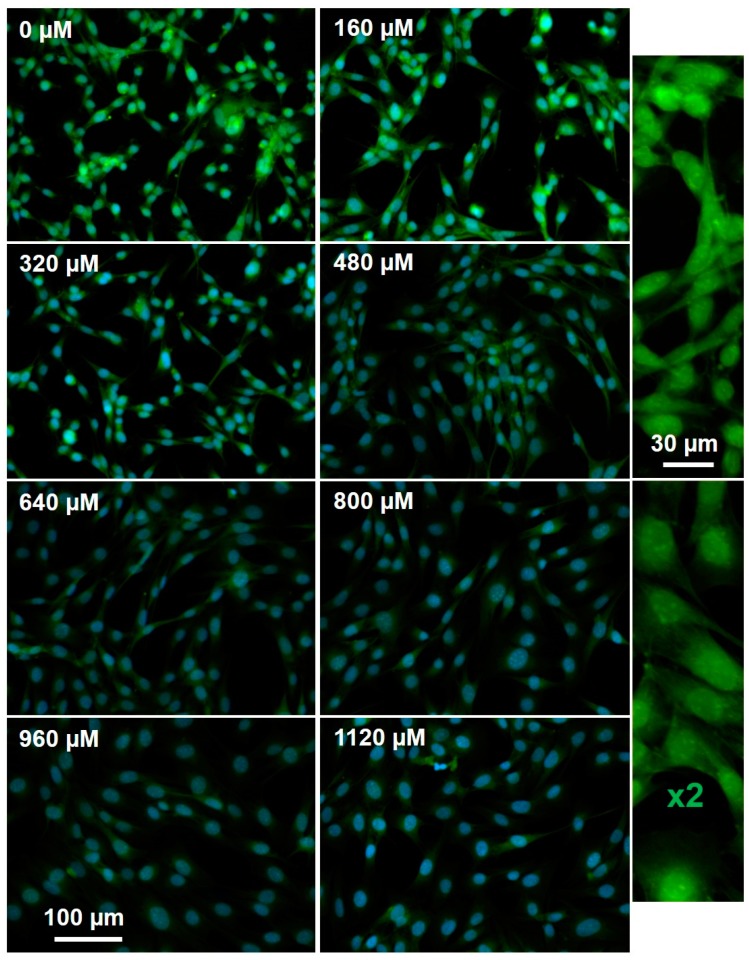
GBM157 cells incubated with increasing (0–1120 µM) concentrations of TMZ and then labeled using 100 µM O^6^PGG with Azido-PEG-FITC and DAPI.

**Figure 4:**
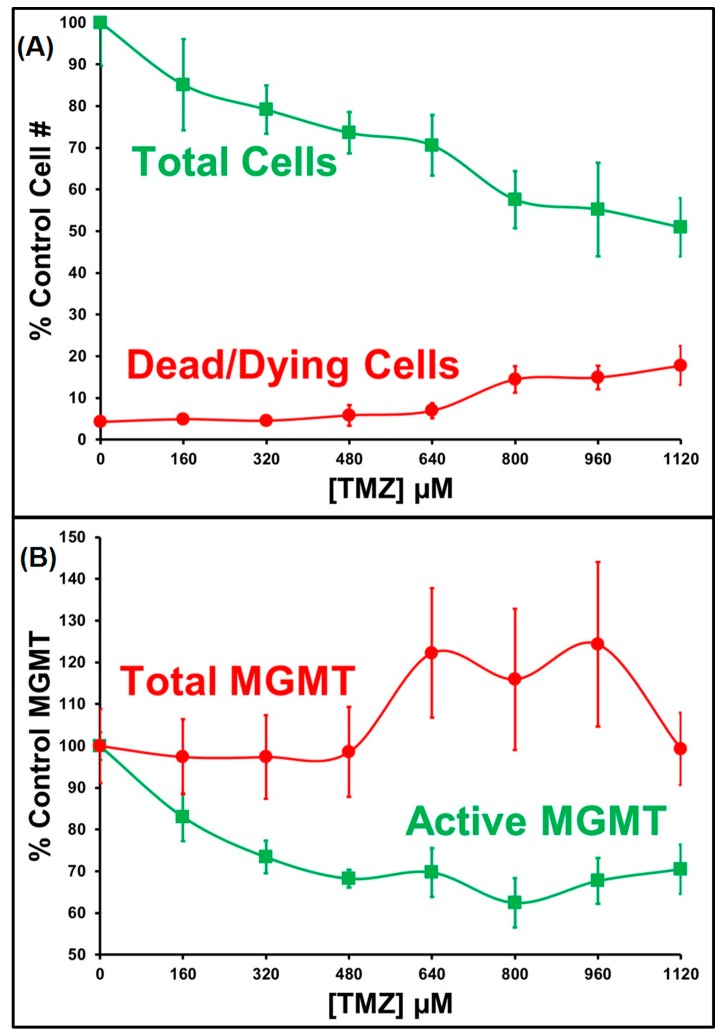
Cell numbers (**A**) and the levels of active and total MGMT (**B**) in TMZ-treated GBM157 cells.

We incubated GBM cells with increasing concentrations of TMZ (Figure 3) followed by labeling with O^6^PGG (100 µM) and visualization with azido-PEG-FITC. Total MGMT-protein levels were determined by labeling with an anti-MGMT antibody and a fluorescently labelled secondary antibody. The levels of both active and total MGMT per cell were quantified by measuring total fluorescence per field after counting nuclei and division (Figure 4).

As expected, fluorescence microscopy showed that increasing TMZ concentrations led to the corresponding reduction in both live cells and active MGMT levels. Higher TMZ levels cause increasing DNA damage, a reduction of the total number of cells and the presence of aberrant nuclei in surviving cells. Active MGMT levels in surviving cells fell and then plateaued at TMZ > 500 µM, and at this inflection point, we were able to measure an increase in the total levels of MGMT protein. 

This suggests either that GBM cells respond to higher levels of DNA damage by upregulating MGMT synthesis or that by incubating with high TMZ concentrations, cells that can be observed are only those which inherently have high levels of MGMT. Further, an analysis of the localization of active MGMT (cytosolic or nuclear) showed that in cells incubated with TMZ, levels of active MGMT fell more in the cytosolic compartment than in the nucleus (see right-hand panels). 

This is somewhat paradoxical as the substrate for the inactivation of MGMT is nuclear DNA damaged by TMZ. In a typical two-compartment model of signal loss of active MGMT, one would expect the loss of active MGMT in the nuclear compartment (because of reaction with O^6^MeG) and a drop in cytosolic levels because of transfer from the cytosol to the nucleus, with the latter always lagging the former. However, the kinetics of loss of active MGMT in the two locales suggest that there is an active transfer process between the nuclear and cytosolic pools of MGMT.

### 2.4. Active MGMT Levels Rapidly Increase at Higher Concentrations of Reactive Oxygen Species 

**Figure 5:**
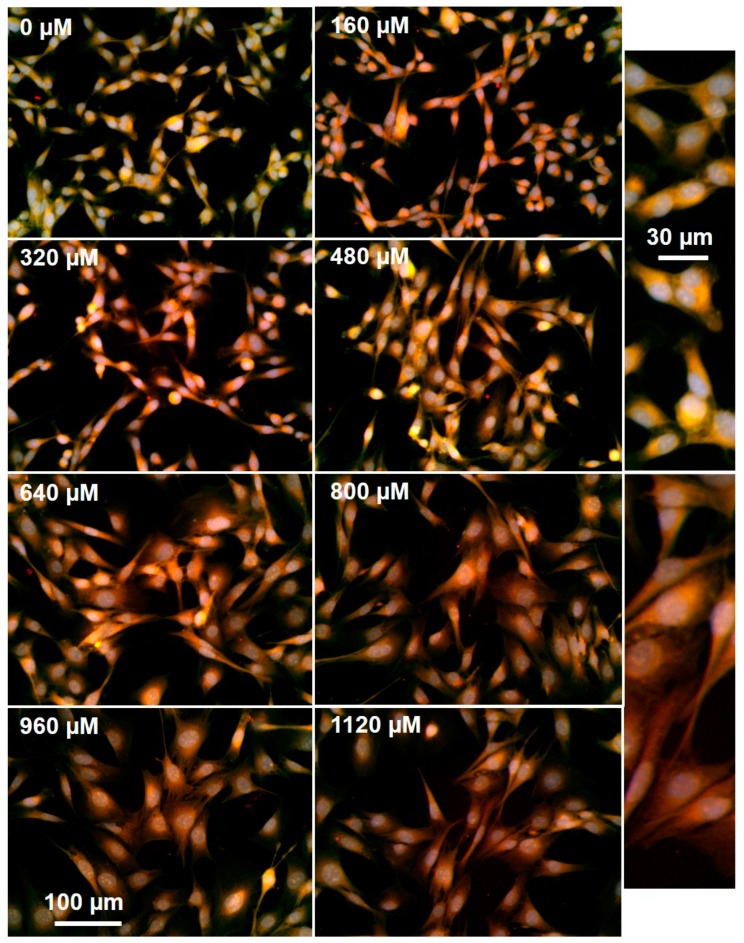
GBM157 cells with increasing (0–1120 µM) concentrations of TMZ, with active MGMT measured using 100 µM O^6^PGG/Azido-PEG-FITC (Green) and Mitosox (Red).

The Keap-Nrf2 pathway modulates cellular responses to redox imbalances, both oxidative and electrophilic, by binding to specific regions of gene promoters (antioxidant response elements, AREs) [22]. Nrf2 controls both basal and induced expression of an array of genes that are involved in detoxification of reactive oxygen species (ROS), including γ-glutamylcysteine synthase, glutathione S-transferase-π, and NAD(P)H oxidoreductase 1. MGMT gene expression is modulated by ROS and is subject to control by Nrf2 via two AREs in this gene’s promoter region. In MCF7 breast cancer cells, the steady-state levels of MGMT are upregulated by about four-fold after Nrf2 levels were elevated using an expression vector [23]. In the same manner, antioxidants like resveratrol cause a drop in MGMT levels, via the NF-kB/Nrf2 dependent pathway, and sensitize cultured GBM cells toward TMZ [24]. To examine the relationship between TMZ induced ROS and active MGMT levels in GBM cells, we incubated cells with the mitochondrial superoxide probe Mitosox and the active MGMT probe O^6^PGG, in the presence of increasing TMZ (Figure 5). As expected, high levels of TMZ corresponded with an increase in Mitosox labeling and a collapse in active MGMT levels. Aberrant enlarged nuclei (stained with DAPI) were also seen at high TMZ concentrations, consistent with increased DNA damage. 

### 2.5. Levels of PCNA and Phosphorylated MGMT Rise in Response to Oxidative stress, but Fall in Response to TMZ Induced DNA Damage 

**Figure 6:**
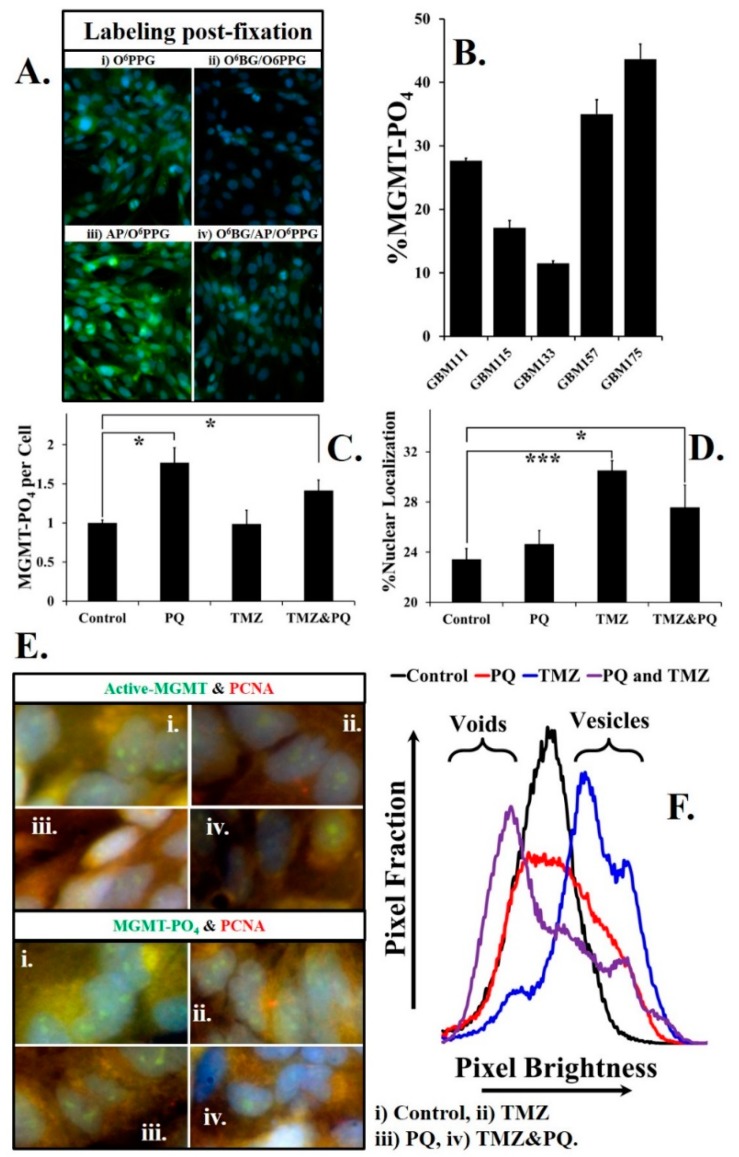
Phosphorylated MGMT in fixed cells can be labeled after reactivation by alkaline phosphatase. (**A**) (i) GBM157 cells labeled using O^6^PGG; (ii) treatment with MGMT inhibitor O^6^BG eliminates the O^6^PGG labeling of MGMT; (iii) pre-treatment of GBM157 cells with alkaline phosphatase (AP) increases MGMT labeling compared to control because of the conversion of phosphorylated MGMT into active MGMT; (iv) reactivation of phosphorylated (inactive) MGMT by AP in cells pre-treated with the MGMT inhibitor O^6^BG. (**B**) The fraction of phosphorylated (inactive) MGMT in different primary GBM cell lines show significant variations, and may reflect tumor heterogeneity; also see Supplementary Figure S3. (**C**) Statistically significant (*t*-test) increases in phosphorylated (inactive) MGMT levels occur upon treating GBM157 cells with either paraquat (PQ), or TMZ, or a combination of PQ and TMZ. (**D**) Statistically significant increases in phosphorylated (inactive) MGMT levels occur upon treating GBM157 cells with TMZ or a combination of PQ and TMZ. (**E**) Proliferating cell nuclear antigen (PCNA) levels were measured using a fluorescently labeled PCNA antibody. Active MGMT levels were measured using O^6^PGG/azido-PEG-FITC/click. Levels of phosphorylated (inactive) MGMT were measured by pre-treating GBM157 cells with O^6^BG followed by treatment with alkaline phosphatase and labeling using O^6^PGG/azido-PEG-FITC/click. (**F**) Analysis of the distribution of PCNA labeled pixels in the GBM157 nuclei. Treatment of GBM157 cells with PQ or TMZ or a combination of PQ and TMZ changes the line shape, indicating higher localization in nuclear vesicles in response to DNA damage.* *p* < 0.05, *** *p* < 0.001

MGMT is subject to site-specific phosphorylation, on a tyrosine residue at the mouth of the substrate channel. Following this (reversible) phosphorylation, the substrate channel is occluded and MGMT is inactivated [25]. We discovered that PFA-fixed, phosphorylated/inactive MGMT could be reactivated and thus become a substrate for O^6^PGG, by incubation with alkaline phosphatase (AP). Figure 6A shows the total active MGMT in fixed GBM cells that are treated with O^6^PGG/azido-PEG-FITC/click (Figure 6A(i)). When the GBM cells were incubated with O^6^BG to remove all enzyme activity, green labeling is low (Figure 6A(ii)). Total phosphorylated/unphosphorylated MGMT can be measured by incubation with AP, before O^6^PGG/azido-PEG-FITC/click (Figure 6A(iii)). Phosphorylated MGMT levels are revealed by incubation with O^6^BG, fixation, and incubation with AP followed by O^6^PGG/azido-PEG-FITC/click (Figure 6A(iv)). We assayed the steady-state levels of phosphorylated MGMT in five different GBM cultures, Figure 6B, and found it varied between 10% and 50% of the total. 

Although ROS can upregulate MGMT levels, via NRF2 [22,23,26,27], it is not known how ROS can alter the phosphorylation status of MGMT. We measured the phosphorylation status of MGMT exposed to the ROS generator paraquat (PQ) and/or TMZ (Figure 6C). Compared to control, a statistically significant increase in phosphorylated MGMT occurs when GBM cells are exposed to PQ and a combination of TMZ and PQ (*p* < 0.05), but not TMZ alone. Interestingly, there is a greater increase in phosphorylated MGMT in cells exposed to TMZ (*p* < 0.001) than in cells exposed to PQ (Figure 6D), presumably reflective of active repair response to DNA damage by TMZ. Statistically significant increase in phosphorylated MGMT also occurs in cells exposed to both PQ and TMZ (*p* < 0.05, Figure 6D). 

Proliferating cell nuclear antigen (PCNA) is a homotrimeric DNA clamp that serves as a scaffold to recruit various proteins involved in DNA replication, repair, remodeling, and epigenetics. Mostofa et al. [28] recently reported the presence of a PCNA interacting protein (PIP box) motif in MGMT and described the serendipitous association of MGMT with PCNA and the cell-cycle inhibitor p21^cip1^ in GBM. They reported that alkylation-induced DNA damage increased the co-localization of MGMT and PCNA. We were interested in determining which form of MGMT—active or inactive—was associated with PCNA and how DNA damage or oxidative stress altered these associations. 

PCNA levels were determined using fluorescently labeled PCNA antibody (PC10 Anti-PCNA antibody, Abcam) while active and inactive MGMT levels were determined as above. Our experiments (Figure 6E,F, and Supplementary Figure S4) show that both active and phosphorylated MGMT are co-localized with PCNA. Exposure to TMZ causes a statistically significant reduction of PCNA (*t*-test, *p* < 0.01) levels, reflecting the recruitment of PCNA for DNA repair. As expected levels of active MGMT were also lowered on exposure to TMZ, however, this was not statistically significant. In contrast, when GBM cells were exposed to oxidative stress by PQ, a statistically significant increase in active MGMT (*p* < 0.01) could be observed (Supplementary Figure S4). 

An analysis of the distribution of bright (PCNA labeled) pixels in the GBM nuclei is shown in Figure 6F. For control cells, the brightness of pixels in the nucleus is normally distributed (Figure 6F, control, black). In cells treated with PQ, the pixel-brightness curve (red) still approximates a normal curve, but is flatter, indicating a wider distribution of PCNA in the nucleus. However, TMZ treatment causes a significant shift toward the right (blue, labeled vesicles), indicating PCNA localization in nuclear vesicles. In cells treated with both PQ and TMZ, an increase in the number of low-intensity pixels compared to brighter pixels can be seen, shifting the curve to the left of the peak seen with control cells (labeled voids). The precise reasons for the shift in PCNA labeling patterns seen above are unclear and may be due to changes in MGMT/PCNA association, or association of PCNA/MGMT with other non-repair proteins upon exposure to oxidative stresses or alkylating DNA damage.

### 2.6. Staining of GBM Microarray Demonstrates Variability in Active and Total MGMT Levels

**Figure 7:**
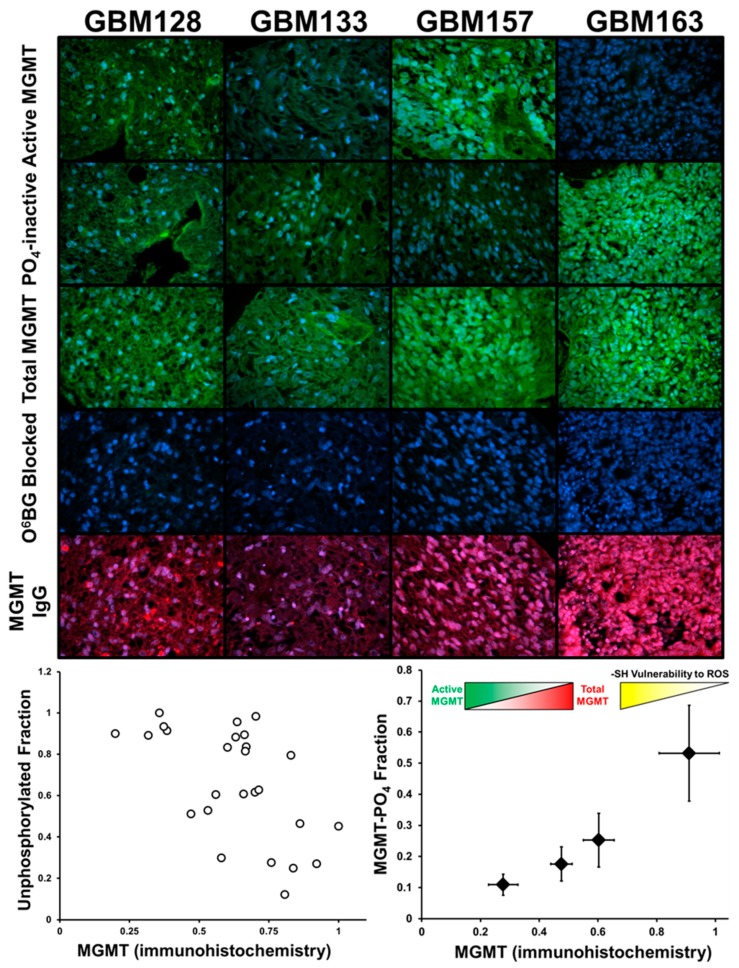
Labeling of MGMT can be done in preserved GBM tumor microarrays after rehydration. Tumor microarrays were rehydrated and then incubated with 100 µM O^6^PGG followed by labeling with Azido-PEG-FITC and Mitosox. Phosphorylated MGMT was labeled by first treating the tumor microarray with O^6^BG to block active MGMT. Washed cells were then treated with alkaline phosphatase and subsequently incubated with 100 µM O^6^PGG and labeled with Azido-PEG-FITC. Total MGMT levels (MGMT IgG) were calculated by immunohistochemical staining using an anti-MGMT antibody.

Figure 7 shows the results from labeling studies performed on a rehydrated 22 GBM tumor microarray, labeled for phosphorylation active/inactive MGMT and total protein, with images from four representative GBM tumors shown. Tumor sections treated with O^6^PGG/azido-PEG-FITC/click show active MGMT levels. Treatment of slices with O^6^BG, then alkaline phosphatase, followed by O^6^PGG/azido-PEG-FITC/click allows visualization of inactive MGMT. Total MGMT activity (phosphorylated/unphosphorylated) uses O^6^PGG/azido-PEG-FITC/click after AP incubation. Background green fluorescence is visualized using O^6^PGG/azido-PEG-FITC/click following O^6^BG. Finally, the labeling of cells with an anti-MGMT antibody was done to examine the total MGMT, independent of its phosphorylation state. Images were obtained for seven fields for each cell line. Pre-treating the cells was done to determine binding at the MGMT active site.

Results from this experiment indicate that the four primary GBM tumors demonstrate significant differences in both the levels and localization of MGMT, reflective of a highly irregular tumor microenvironment and local oxidative stresses experienced by the tumors. For instance, the GBM163 tumor slice showed very low levels of active MGMT but very high levels of the inactive, phosphorylated MGMT. In contrast, GBM157 cells show high levels of active MGMT but low levels of phosphorylated MGMT. The other two tumors, GBM128 and GBM133 have intermediate levels of active and phosphorylated MGMT. 

The wide variability in MGMT levels and post-translational modification of 22 different GBM tumor slices can also be observed by comparing MGMT phosphorylation to total MGMT levels (Figure 7 bottom left). Dividing phosphorylated MGMT levels into quartiles and plotting against MGMT levels measured by antibody level indicates that high levels of ROS-insensitive, phosphorylated MGMT are present when total MGMT is high (Figure 7, bottom right). Phosphorylation of MGMT has been shown to prolong its cellular half-life by shielding the reactive Cys145 thiol from oxidative inactivation by ROS. Consequently, GBM cells with a higher fraction of its total MGMT in the inactive form would be more resistant to ROS-mediated inactivation of MGMT. These cells would also have higher reserves of MGMT to readily tap into when challenged by DNA damaging alkylating agents and would be expected to be more resistant to such agents. In contrast, GBM cells with a lower fraction of total MGMT in the phosphorylated form would be expected to have greater sensitivity to ROS and be deficient in their response to DNA damage. 

## 3. Discussion

### Active MGMT Levels are Finely Tuned and Responsive to Levels of DNA Damage

We propose a simple model for the protection of MGMT from ROS and the direction of active MGMT into the nucleus.

**Figure 8:**
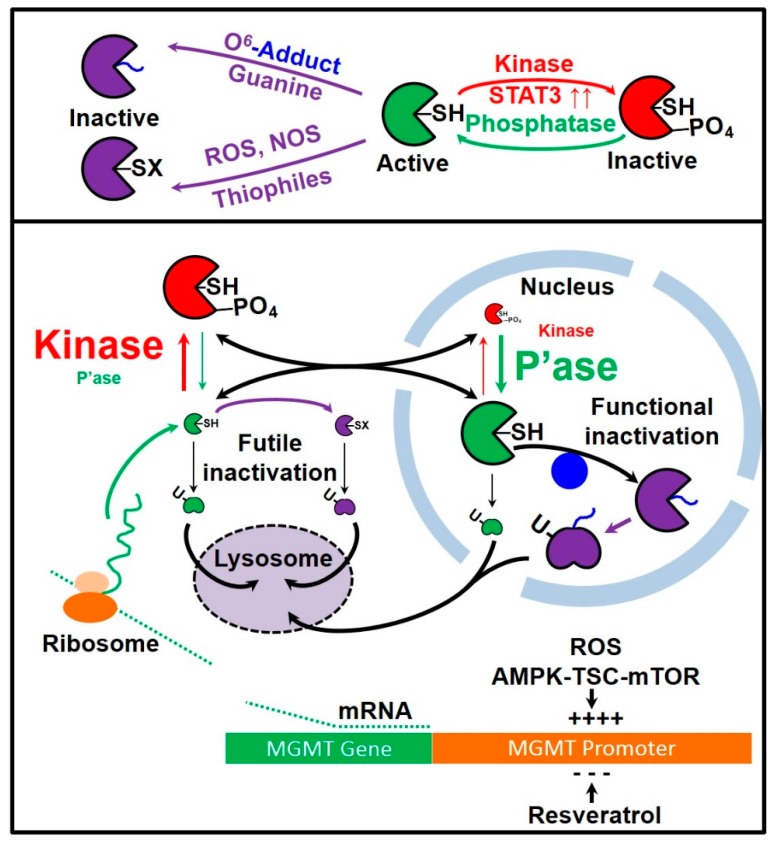
MGMT lifecycle in cancer cells. Figure key: PO_4_—phosphate group; -SH—thiol group of Cys145 in MGMT; -SX—inactivated MGMT; P’ase—phosphatase; U—ubiquitin. Phosphorylation of active MGMT functionally inactivates it. Active MGMT can be regenerated by the action of intracellular phosphatases. MGMT can also be inactivated by its actions to remove DNA O^6^-adducts (functional inactivation) or by reaction with reactive oxygen species or other cellular electrophiles (futile inactivation). Inactive MGMT is ubiquitinated and is transported for degradation in lysosomes.

The use of the suicide enzyme MGMT by cells to remove lethal O^6^-alkyl G lesions presents cells with a problem. MGMT is synthesized in the cytosol and may undergo futile deactivation in the cytosol by reactive species such as ROS before it can migrate to the nucleus. ROS can react with Cys145 of MGMT and deactivate it. Exposure of cells to methylation agents increase ROS levels (Figure 8). However, in TMZ titrations, we observe more active MGMT in the nucleus than in cytosol and observe biphasic kinetics for the loss of active MGMT. 

We suggest that MGMT phosphorylation is a means to close the substrate channel, protecting the Cys145 thiol from oxidation by ROS, thus arresting futile oxidative inactivation. We further suggest that the ratios of the MGMT kinase/phosphatase are inverted in the cytosolic/nuclear compartments. In the cytosol phosphorylated MGMT levels are high, as kinase activity > phosphatase activity, and in the nucleus, the opposite is true, with phosphorylated MGMT levels low, as phosphatase activity > kinase activity. The positive correlation between levels of phosphorylated MGMT and total MGMT suggests that the promoters of the putative MGMT kinase and the putative MGMT phosphatase would be downstream of Nrf2-controlled AREs in their promoter regions. 

Taken together, our experiments suggest that steady-state levels of active MGMT in cells are achieved by a finely tuned balance between MGMT production in response to DNA damage and/or oxidative stress and degradation of inactivated ubiquitinated MGMT. From a cellular perspective, generating MGMT (a single-use suicide enzyme with a highly reactive thiol moiety) is an expensive proposition. Additionally, while MGMT is synthesized in cytosolic ribosomes, its primary site of action is in the nuclear DNA. Once synthesized, MGMT is phosphorylated into an inactive phosphorylated form that has a long half-life [25]. Inactivation of MGMT by phosphorylation shields the reactive thiol in MGMT from futile inactivation within the cytosol. Upon diffusion into the nucleus, phosphorylated MGMT is converted to its active form by the action of phosphatases. This would imply that the steady-state levels of active MGMT are determined by the ratio of kinase/phosphatase activity, thus providing a finely-tunable means to control the activity of this expensive DNA-repair enzyme. 

## 4. Materials and Methods 

Amino-PEG_5000_-azide trifluoroacetate salt was obtained from JenKem Technologies, China. All other reagents were obtained from Sigma-Aldrich, USA, and used without further purification. Mass spectra were determined on a Thermo-Scientific LCQ FLEET spectrometer in electron impact (EI) mode. Proton (^1^H) and carbon (^13^C) nuclear magnetic resonance (NMR) spectra were recorded at 600 and 150 MHz, respectively, on a Bruker spectrometer employing the solvent as an internal standard. Spectra were recorded in DMSO-d6 as the solvent, and *J* values are given in hertz (Hz). NH signals appeared as broad singlets (br s) exchangeable with D_2_O.

TLCs were done using Merck glass sheets precoated with Kieselgel 60F_254_ (0.2 mm) as the adsorbent and were visualized with UV light at 254 and 365 nm. Column chromatography was conducted under medium pressure on silica (Kieselgel 60, 240–400 mesh). 

O^6^-Propargylguanine was synthesized as reported in the literature [21]. Propargyl alcohol (0.17 mL, 2.95 mmol, 2.5 mol equiv) was dissolved in anhydrous THF (5 mL) and cooled to 0 ^o^C in an ice bath. A solution of sodium hydride (94 mg, 2.36 mmol, 2 mol equiv, 60% dispersion mineral oil) in dry THF (5 mL) was added to this cooled solution and the mixture was stirred for 30 min at 0 ^o^C. 2-Amino-6-chloropurine (200 mg, 1.18 mmol, 1.0 mol equiv) was added and the mixture was heated to reflux under N_2_ until TLC analysis confirmed consumption of starting material. The reaction mixture was neutralized with glacial acetic acid, and the solvent was removed in vacuo. The product was purified by chromatography on silica (EtOAc: EtOH 9:1) and recrystallization from ethanol and was obtained as a white solid.

^1^H NMR δ 3.59 (1H, t, *J* = 2.4, CH), 5.11 (2H, d, *J* = 2.4, CH_2_), 6.34 (2H, s, NH_2_), 6.56 (1H, s, 8-H); MS (EI) *m*/*z* 190.02, (M^+^H) calcd for C_8_H_7_N_5_O 189.0638.

Amino-PEG_5000_-azide trifluoroacetate salt (50 mg, ~0.01 mM) was added to fluorescein isothiocyanate (10 mg, 0.025 mM) and *N*,*N-*diisopropyl ethylamine (200 µL) in 5 mL anhydrous DMF and stirred overnight while protected from light. The resulting dark orange reaction mixture was purified by dialysis against 1000 mL deionized water using a dialysis membrane with 0.5-1 kDa MWCO for 24 h followed by lyophilization. Further concentrated by ultrafiltration (Millipore filter packs, MWCO = 3000) centrifuged at 4000 rpm for 6 h, dilution of the concentrate with 1 mL with deionized water, re-centrifugation at 4000 rpm for 6 h and lyophilization gave azido-PEG-FITC as a sticky dark orange solid that could be readily dissolved in water. The MALDI-ESI spectra for azido-PEG-FITC and the starting material, amino-PEG_5000_-azide are provided in Supplementary Figure S5A,B respectively.

Permission: Glial tumors and the cultures derived from them were obtained from de-identified patient tissues and have no private information identifiable under IRB Protocol 00014547.

Tumor samples and cell cultures: GBM tumor tissue was harvested at the time of surgical excision and assigned a laboratory ID (GBMXXX). The tissue was then washed in PBS (Fisher Scientific, Waltham, MA). Portions were minced with a scalpel, homogenized, and grown in Dulbecco’s modified Eagle’s medium (DMEM) with fetal bovine serum (FBS, 20%), 1U GlutaMax™, sodium pyruvate (1mM), penicillin (100 U/mL), and streptomycin (100 mg/mL). All GBMXXX cells are spontaneously immortal and primary stocks were frozen at the fourth or fifth passage and used between the seventh and ninth passages. GBM157 and GBM175 cells were spontaneously immortal, so they were frozen at the fourth passage and used between sixth and ninth passages.

Staining of cells: GBM cells were incubated 10 µM O^6^BG, an MGMT inhibitor, for varying durations, and then 100 µM O^6^PGG was added. After a further incubation of 5 min, cells were fixed with paraformaldehyde (PFA). The cells were washed and permeabilized, and azido-PEG-FITC dye (~100 µM) was added along with 100 µM copper sulfate, 500 μM THPTA ligand, and 5 mM ascorbate, typically overnight. Cells were then repeatedly washed to remove any free dye and then visualized using fluorescence microscopy. The nuclei were stained with DAPI.

### 4.1. Fluorescence Microscopy

Images were captured using a Nikon Eclipse TE2000-E at 4×, 20×, or 30× magnification using a CoolSnap ES digital camera system (Roper Scientific) containing a CCD-1300-Y/HS 1392-1040 imaging array that is cooled by Peltier. Images were recorded and analyzed using Nikon NIS-Elements software (Elements 3.22.11). All images were saved as JPEG2000 files using Nikon NIS-Elements. The emission of FITC-labeled MGMT was collected using ex 450–490 nm, em 500–550 nm and Hoechst 33342 using ex 325–375 nm, em 435–485 nm.

GBM cells were incubated for 30 min with Hoechst 33342 (10 μM) and then were fixed with ice-cold 4% paraformaldehyde. Cell counts were conducted in the center field at ×4 or ×20 magnification, depending on cell density. Dead/dying cells were identified as having condensed nuclei with signal intensities over three-fold that of the median cell nuclei or being identified as fragmented.

GBM tissue microarray blocks were sliced into 5μm sections and were affixed to slides and dried. Slides were de-waxed (four times) in xylene, twice in isopropanol, and rehydrated using graded ethanol [29]. 

### 4.2. Measurement of Active MGMT Levels

Active MGMT was labeled by incubating cells with 100 μM O^6^PGG for 10 min, before fixation. The copper-catalyzed azide-alkyne cycloaddition was performed in 100 μM CuSO4, 500 μM THPTA ligand, 5 mM ascorbate and 10 μM FITC-azido PEG, typically overnight at room temperature.

MGMT protein levels were visualized using a mouse monoclonal IgG (E-1) from Santa Cruz Biotechnology as primary and an Alexa-594 goat anti-mouse secondary.

## 5. Conclusions

The method we present offers a simple and effective method to measure the levels of active MGMT in cancer cells. These studies demonstrate an increase in levels of active MGMT in response to DNA damage by alkylating agents. Analysis of active MGMT levels in response to increasing TMZ concentrations indicates that active MGMT follows biphasic kinetics with buffering capacity afforded by ready conversion of inactive phosphorylated MGMT by putative phosphatases into its active form. This study also reiterates the role played by MGMT to protect cells from genotoxic DNA-alkylation events. 

## Supplementary Materials

The following are available online at https://www.mdpi.com/2072-6694/12/2/453/s1. Figure S1: Nuclear co-localization with O^6^BG. Figure S2: Measuring PCNA and MGMT levels upon treatment with PQ or TMZ. Figure S3: MALDI spectra of azido-PEG-FITC (Figure S3A) and the starting material (Figure S3B).

## References

[1] Gerson S.L. (2004). MGMT: Its Role in Cancer Aetiology and Cancer Therapeutics. Nature Rev. Cancer.

[2] Zhang J., Stevens M.F.G., Bradshaw T.D. (2012). Temozolomide: Mechanisms of Action, Repair and Resistance. Curr. Mol. Pharmacol..

[3] Warren J.J., Forsberg L.J., Beese L.S. (2006). The structural basis for the mutagenicity of O^6^-methyl-guanine lesions. Proc. Natl. Acad. Sci. USA.

[4] Stupp R., Mason W.P., van den Bent M.J., Weller M., Fisher B., Taphoorn M.J.B., Belanger K., Brandes A.A., Marosi C., Bogdahn U. (2005). Radiotherapy plus Concomitant and Adjuvant Temozolomide for Glioblastoma. N. Engl. J. Med..

[5] Batchelor T.T., Mulholland P., Neyns B., Nabors L.B., Campone M., Wick A., Mason W., Mikkelsen T., Phuphanich S., Ashby L.S. (2013). Phase III randomized trial comparing the efficacy of cediranib as monotherapy, and in combination with lomustine, versus lomustine alone in patients with recurrent glioblastoma. J. Clin. Oncol..

[6] Taal W., Oosterkamp H.M., Walenkamp A.M., Dubbink H.J., Beerepoot L.V., Hanse M.C., Buter J., Honkoop A.H., Boerman D., de Vos F.Y. (2014). Single-agent bevacizumab or lomustine versus a combination of bevacizumab plus lomustine in patients with recurrent glioblastoma (BELOB trial): A randomised controlled phase 2 trial. Lancet. Oncol..

[7] Sharpe M.A., Raghavan S., Baskin D.S. (2018). PAM-OBG: A monoamine oxidase B specific prodrug that inhibits MGMT and generates DNA interstrand crosslinks, potentiating temozolomide and chemoradiation therapy in intracranial glioblastoma. Oncotarget.

[8] Cabrini G., Fabbri E., Lo Nigro C., Dechecchi M.C., Gambari R. (2015). Regulation of expression of O^6^-methylguanine-DNA methyltransferase and the treatment of glioblastoma (Review). Int. J. Oncol..

[9] Hazra T.K., Roy R., Biswas T., Grabowski D.T., Pegg A.E., Mitra S. (1997). Specific recognition of O^6^-methylguanine in DNA by active site mutants of human O^6^-methylguanine-DNA methyltransferase. Biochemistry.

[10] Srivenugopal K.S., Yuan X.H., Friedman H.S., Ali-Osman F. (1996). Ubiquitination-dependent proteolysis of O^6^-methylguanine-DNA methyltransferase in human and murine tumor cells following inactivation with O^6^-benzylguanine or 1,3-bis(2-chloroethyl)-1-nitrosourea. Biochemistry.

[11] Fan C.H., Liu W.L., Cao H., Wen C., Chen L., Jiang G. (2013). O^6^-methylguanine DNA methyltransferase as a promising target for the treatment of temozolomide-resistant gliomas. Cell Death Dis..

[12] Everhard S., Tost J., Abdalaoui H.E., Crinière E., Busato F., Marie Y., Gut I.G., Sanson M., Mokhtari K., Laigle-Donadey F. (2009). Identification of regions correlating MGMT promoter methylation and gene expression in glioblastomas. Neuro-Oncology.

[13] Weller M., Stupp R., Reifenberger G., Brandes A.A., van den Bent M.J., Wick W., Hegi M.E. (2009). MGMT promoter methylation in malignant gliomas: Ready for personalized medicine?. Nat. Rev. Neurol..

[14] Nagel G., Brenner W., Johnsson K., Kaina B. (2003). DNA repair protein O^6^-methylguanine-DNA methyltransferase in testis and testicular tumors as determined by a novel nonradioactive assay. Anal. Biochem..

[15] Yeager N.D., Dolan M.E., Gastier J.M., Gross T.G., Delaney S., Frick J., Ruymann F.B., Ewesuedo R. (2003). O^6^-methylguanine-DNA methyltransferase activity and promoter methylation status in pediatric rhabdomyosarcoma. J. Pediat. Hematol. Oncol..

[16] Encell L.P., Loeb L.A. (1999). Redesigning the substrate specificity of human O^6^-alkylguanine-DNA alkyltransferase. Mutants with enhanced repair of O(4)-methylthymine. Biochemistry.

[17] Kreklau E.L., Limp-Foster M., Liu N., Xu Y., Kelley M.R., Erickson L.C. (2001). A novel fluorometric oligonucleotide assay to measure O-6-methylguanine DNA methyltransferase, methylpurine DNA glycosylase, 8-oxoguanine DNA glycosylase and abasic endonuclease activities: DNA repair status in human breast carcinoma cells overexpressing methylpurine DNA glycosylase. Nucleic Acids Res..

[18] Beharry A.A., Nagel Z.D., Samson L.D., Kool E.T. (2016). Fluorogenic Real-Time Reporters of DNA Repair by MGMT, a Clinical Predictor of Antitumor Drug Response. PLoS ONE.

[19] Tintoré M., Aviñó A., Ruiz F.M., Eritja R., Fàbrega C. (2010). Development of a Novel Fluorescence Assay Based on the Use of the Thrombin-Binding Aptamer for the Detection of O^6^-Alkylguanine-DNA Alkyltransferase Activity. J. Nucl. Acids.

[20] Robinson C., Palomo J., Vogelbaum M.A. (2010). Thin layer chromatography-based assay of O^6^-methylguanine-DNA methyltransferase activity in tissue. Anal. Biochem..

[21] Griffin R.J., Arris C.E., Bleasdale C., Boyle F.T., Calvert A.H., Curtin N.J., Dalby C., Kanugula S., Lembicz N.K., Newell D.R. (2000). Resistance-Modifying Agents. 8. Inhibition of O^6^-Alkylguanine-DNA Alkyltransferase by O^6^-Alkenyl-, O^6^-Cycloalkenyl-, and O^6^-(2-Oxoalkyl)guanines and Potentiation of Temozolomide Cytotoxicity in Vitro by O^6^-(1-Cyclopentenylmethyl)guanine. J. Med. Chem..

[22] Kansanen E., Kuosmanen S.M., Leinonen H., Levonen A.-L. (2013). The Keap1-Nrf2 pathway: Mechanisms of activation and dysregulation in cancer. Redox Biol..

[23] Paranjpe A., Bailey N.I., Konduri S., Bobustuc G.C., Ali-Osman F., Yusuf M.A., Punganuru S.R., Madala H.R., Basak D., Mostofa A. (2016). New insights into estrogenic regulation of O(6)-methylguanine DNA-methyltransferase (MGMT) in human breast cancer cells: Co-degradation of ER-alpha and MGMT proteins by fulvestrant or O(6)-benzylguanine indicates fresh avenues for therapy. J. Biomed. Res..

[24] Huang H., Lin H., Zhang X., Li J. (2012). Resveratrol reverses temozolomide resistance by downregulation of MGMT in T98G glioblastoma cells by the NF-κB-dependent pathway. Oncol. Rep..

[25] Mullapudi S.R., Ali-Osman F., Shou J., Srivenugopal K.S. (2000). DNA repair protein O^6^-alkylguanine-DNA alkyltransferase is phosphorylated by two distinct and novel protein kinases in human brain tumour cells. Biochem. J..

[26] Marinho H.S., Real C., Cyrne L., Soares H., Antunes F. (2014). Hydrogen peroxide sensing, signaling and regulation of transcription factors. Redox Biol..

[27] Reuter S., Gupta S.C., Chaturvedi M.M., Aggarwal B.B. (2010). Oxidative stress, inflammation, and cancer: How are they linked?. Free Radical Biol. Med..

[28] Mostofa A., Punganuru S.R., Madala H.R., Srivenugopal K.S. (2018). S-phase Specific Downregulation of Human O^6^-Methylguanine DNA Methyltransferase (MGMT) and its Serendipitous Interactions with PCNA and p21(cip1) Proteins in Glioma Cells. Neoplasia.

[29] Sharpe M.A., Baskin D.S. (2016). Monoamine oxidase B levels are highly expressed in human gliomas and are correlated with the expression of HiF-1α and with transcription factors Sp1 and Sp3. Oncotarget.

