# Supplementary material for: A ”Clickable” Probe for Active MGMT in Glioblastoma Demonstrates Two Discrete Populations of MGMT
Source: Cancers (Basel). 2020 Feb 14;12(2):453. doi: 10.3390/cancers12020453 (PMC7072665; doi:10.3390/cancers12020453)
Supplement: Supplementary file 1 [file cancers-12-00453-s001.zip › cancers-708168-supplementary.docx]

**Use of a ‘clickable’ probe for active MGMT in glioblastoma demonstrates two discrete populations of MGMT**

**Authors**

Sudhir Raghavan*, David S. Baskin, and Martyn A. Sharpe*

Department of Neurosurgery

Kenneth R. Peak Brain and Pituitary Tumor Center

Houston Methodist Hospital

6565 Fannin Street

Houston, TX 77030

Corresponding author: [sraghavan@houstonmethodist.org](mailto:sraghavan@houstonmethodist.org); [masharpe@houstonmethodist.org](mailto:masharpe@houstonmethodist.org)

**Supplementary information**

**Fig. S1.** Nuclear co-localization with O^6^BG


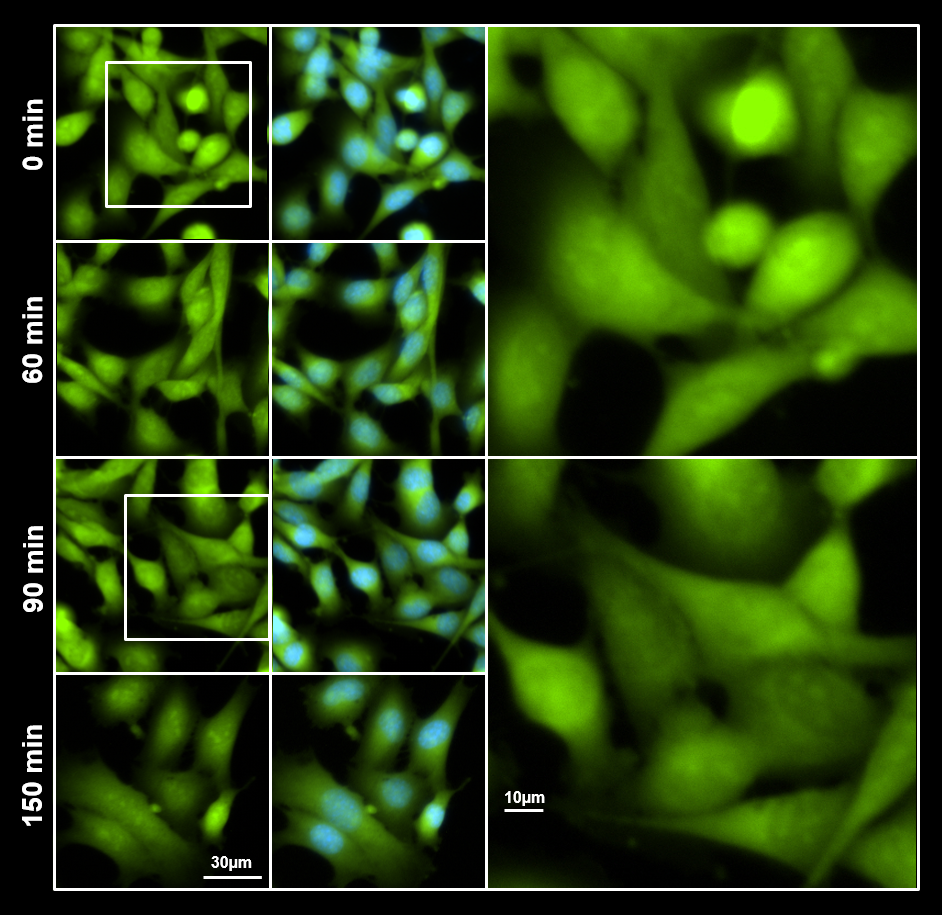


**Fig S1**. MGMT incubated with O^6^PGG and then labeled using azido-PEG-FITC show co-localization in the nucleus (stained by DAPI). Nuclei of cells shown in the enlarged images show MGMT labeled in clusters in the nuclei.

**Fig. S2.** Labeling of MGMT in five primary GBM cell lines

**
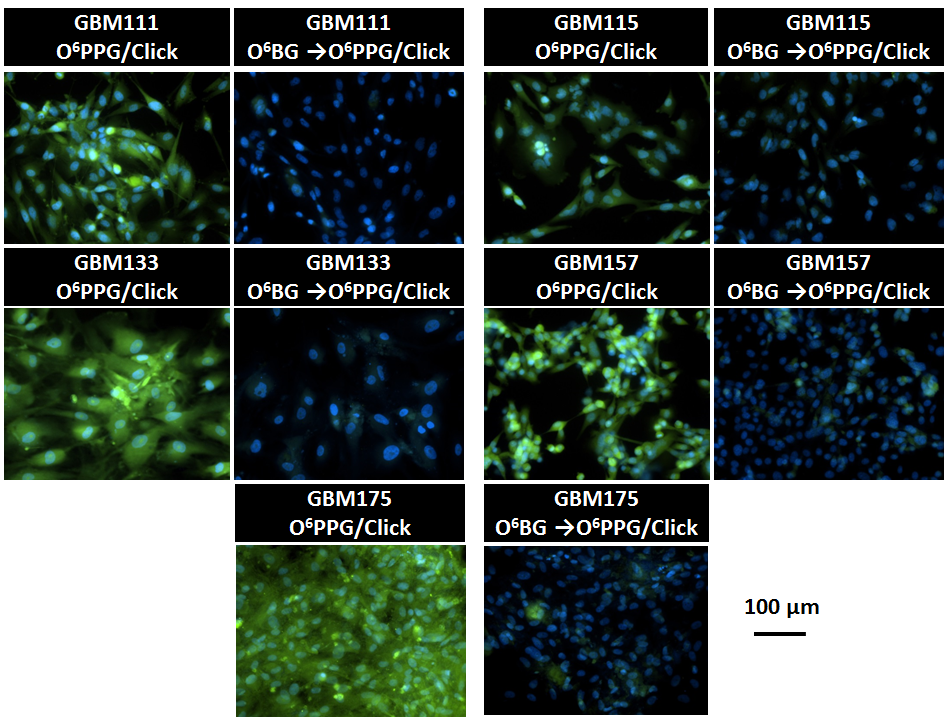
**

**Fig S2**. Five primary GBM cell lines (GBM111, GBM115, GBM133, GBM157 and GBM175) were incubated with O^6^PGG and then labeled using azido-PEG-FITC. Nuclei were stained using DAPI. Significant variations in levels of MGMT can be observed among the different GBM cell lines, reflective of tumor heterogeneity. Labeling of MGMT by O^6^PGG/ azido-PEG-FITC is almost completely inhibited by pre-treating GBM cells with 10 μM O^6^BG before labeling with O^6^PGG/azido-PEG-FITC.

**Fig. S3.** Reactivation of phosphorylated (inactive) MGMT in GBM by treatment with alkaline phosphatase.

**
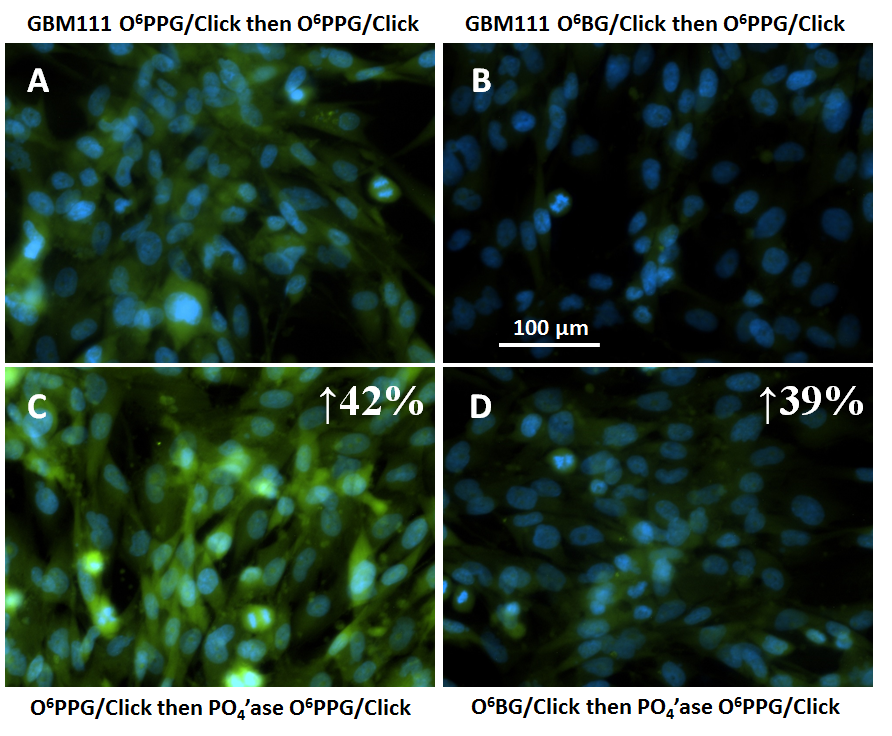
**

**Fig S3**. (A) MGMT is labeled in green in the primary GBM line (GBM111) by incubating cells with O^6^PGG followed by azido-PEG-FITC/click. (B) Pretreating the GBM111 cells with 10 μM O^6^BG before labeling with O6PGG/azido-PEG-FITC significantly reduces MGMT labeling. (C) The fraction of inactive (phosphorylated) MGMT in GBM111 can be observed by treating cells with alkaline phosphatase followed by labeling with O6PGG/azido-PEG-FITC (42% increase in signal compared to (A)). (D) A similar increase (39%, compared to (B)) in signal could be observed when O^6^BG treated GBM111 cells were treated with alkaline phosphatase followed by labeling with O6PGG/azido-PEG-FITC.

**Fig. S4.** Measuring PCNA and MGMT levels upon treatment with PQ, TMZ or PQ and TMZ.


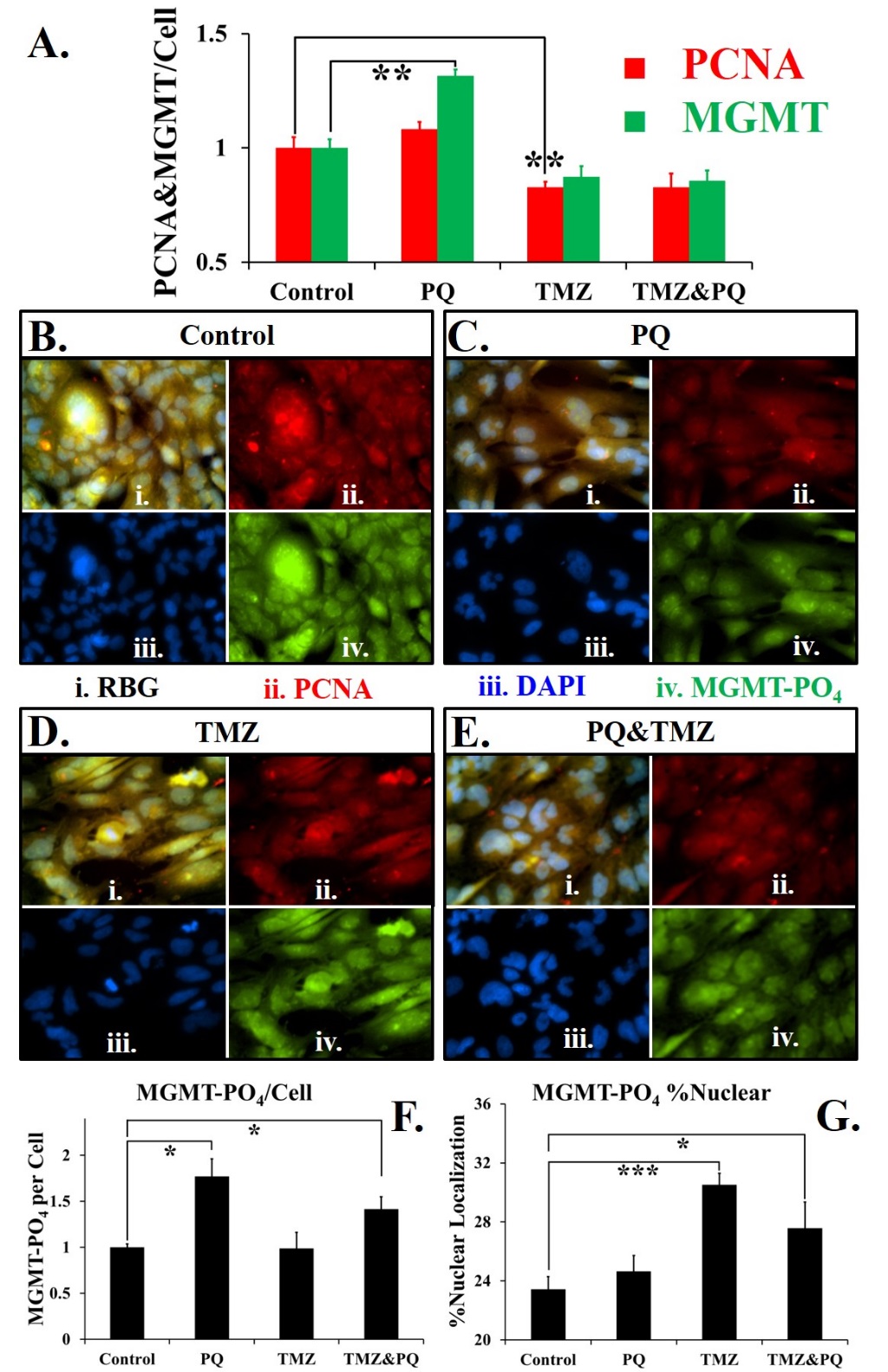


**Fig. S4.** (A) PCNA (red) and MGMT (green) levels increase when GBM cells are exposed to ROS generator paraquat (PQ), but are lowered when cells are exposed to TMZ or a combination of PQ and TMZ. (B)-(D) show (i) combined RBG image of cells labeled with the PCNA antibody (red), DAPI (blue) and FITC labeled phosphorylated MGMT (green). (ii) PCNA labeling of GBM cells. (iii) Nuclear labeling with DAPI; and (iv) labeling of phosphorylated MGMT using O^6^PGG and azido-PEG-FITC in cells treated with O^6^BG and then alkaline phosphatase.

**Fig S5.** MALDI spectra of azido-PEG-FITC (Fig S5A) and the starting material (Fig S5B).

MALDI spectra were obtained on a Waters MALDI SYNAPT machine using cyano-4-hydroxycinnamic acid as the matrix material. Samples were prepared by dissolving azido-PEG_FITC or PEG_5000_-azide in methanol. Masses were measured from 0-6500 Da using a laser energy setting of 300.

**Fig S5A**. MALDI spectrum for azido-PEG-FITC

**Fig S5B**. MALDI spectrum for PEG_5000_-azide
